# Ozone Therapy as a Redox-Modulating Strategy in Degenerative Musculoskeletal Diseases: A Clinical–Mechanistic Review

**DOI:** 10.3390/jcm15156065

**Published:** 2026-08-04

**Authors:** Emma Borrelli

**Affiliations:** Neurosurgery Unit, Public Outpatient Clinic for Ozone Therapy, University Hospital Santa Maria alle Scotte, 53100 Siena, Italy; borrelli@unisi.it

**Keywords:** hormesis, musculoskeletal diseases, Nrf2 pathway, oxidative stress, ozone therapy, redox modulation

## Abstract

**Background/Objectives:** Degenerative musculoskeletal diseases represent a major cause of chronic pain and disability worldwide and are increasingly associated with dysregulated oxidative stress and impaired redox signaling. Conventional therapeutic strategies are often limited to symptomatic management, prompting interest in adjunctive approaches that target underlying biological mechanisms. Among these, medical ozone therapy has been proposed as a redox-modulating intervention in various musculoskeletal conditions. **Methods:** A narrative clinical–mechanistic review of the literature was conducted using PubMed/MEDLINE, Scopus, and Google Scholar to identify experimental and clinical studies examining oxidative stress, redox signaling, and the application of medical ozone therapy in degenerative musculoskeletal disorders. Preclinical models published between 2000 and 2025 and randomized controlled trials published between 2020 and 2025 were critically evaluated and synthesized qualitatively, with attention to biological plausibility, clinical outcomes, and methodological limitations. A PRISMA-style flow diagram was used to document study selection. **Results:** Evidence from experimental studies supports a role for controlled oxidative stimuli in activating adaptive cellular defense pathways, including redox-sensitive signaling mechanisms involved in inflammation and tissue homeostasis. Clinical studies across conditions such as intervertebral disc disease, knee osteoarthritis, and other degenerative or overuse-related disorders report short- to mid-term improvements in pain and function following ozone therapy. However, substantial heterogeneity in study design, treatment protocols, and outcome measures limits comparability. Critically, many trials report statistically significant differences that do not consistently exceed the minimal clinically important difference (MCID) for pain and disability indices, and no trial has validated the proposed Nrf2-mediated mechanism in human tissue at clinically administered doses. **Conclusions:** Current evidence suggests that ozone therapy may exert biologically plausible effects through hormetic redox modulation and may provide symptomatic benefit in selected patients with degenerative musculoskeletal diseases. Nonetheless, the lack of standardized protocols, high-quality long-term data, and mechanistic biomarker validation warrants cautious interpretation. Ozone therapy should be regarded as an investigational adjunct rather than a disease-modifying or standalone standard of care. Further rigorous clinical trials integrating mechanistic biomarkers, sham-controlled designs, and standardized methodologies are needed to clarify the therapeutic role and safety profile of ozone therapy in musculoskeletal medicine.

## 1. Introduction

Degenerative musculoskeletal diseases, including intervertebral disc degeneration and osteoarthritis, are among the leading causes of chronic pain, disability, and reduced quality of life worldwide. Traditionally interpreted as the inevitable consequence of mechanical overload and aging, these conditions are now increasingly recognized as biologically active disorders characterized by progressive alterations in cellular functions and metabolic homeostasis rather than purely structural wear [1,2]. Accumulating experimental and clinical evidence indicates that chronic oxidative stress caused by an increasing amount of reactive oxygen species (ROS), in combination with mitochondrial dysfunction and persistent low-grade inflammation, may play a role in the pathogenesis and progression of musculoskeletal degeneration. In fact, it has been reported that in cartilage, intervertebral discs, and periarticular tissues, sustained redox imbalance contributes to extracellular matrix degradation, impaired anabolic signaling, cellular senescence, and reduced tissue repair capacity. These processes promote structural deterioration and symptom persistence, particularly in chronic and recurrent disease courses [3,4]. Importantly, ROS are no longer viewed solely as cytotoxic by-products of metabolism: at physiological concentrations, they act as intracellular signaling molecules regulating redox-sensitive transcription factors and adaptive pathways involved in cellular defense, energy metabolism, and inflammatory control. Thus, disease progression appears to reflect not only cumulative oxidative damage, but also the failure of endogenous redox-regulatory mechanisms that normally restore tissue homeostasis [5].

Based on this evidence, in chronic oxidative stress diseases, therapeutic strategies aimed at restoring redox balance through the activation of endogenous adaptive responses have gained increasing interest. This concept differs fundamentally from conventional approaches focused on suppressing inflammation or administering exogenous antioxidants, which may not adequately address impaired redox signaling and, in some contexts, may interfere with physiological adaptive processes [6,7]. As a consequence of these changes in therapeutic perspectives of chronic oxidative pathologies, the use of ozone in medicine has been suggested as a conservative intervention able to modulate redox homeostasis through a controlled and transient oxidative stimulus. According to several experimental works, ozone as a therapeutic agent, when administered at low and calculated doses, rapidly reacts with biological substrates, generating intracellular secondary messengers able to activate cytoprotective and antioxidant pathways. However, despite growing empirical use in chronic diseases including musculoskeletal disorders, the role of ozone therapy remains controversial due to heterogeneity in treatment protocols, study designs, and outcome measures [8].

The present clinical review examines the biological rationale for ozone therapy within the context of redox biology and summarizes current experimental and clinical evidence regarding its application in degenerative musculoskeletal diseases. By adopting a disease-oriented and mechanistic perspective, the aim of this review is to clarify potential clinical indications, highlight methodological limitations, and identify priorities for future research.

## 2. Methods: Literature Search and Study Selection

A comprehensive literature review was conducted to identify experimental and clinical studies addressing oxidative stress, redox signaling, and the biological rationale and clinical applications of medical ozone therapy in degenerative musculoskeletal diseases. The review aimed to provide a critical and up-to-date synthesis of available evidence while accounting for heterogeneity in study design, interventions, and outcomes.

Electronic searches were performed in PubMed/MEDLINE, Scopus, and Google Scholar using a combination of Medical Subject Headings (MeSH) terms and free-text keywords, including “ozone therapy,” “medical ozone,” “oxidative stress,” “redox signaling,” “hormesis,” “musculoskeletal degeneration,” “osteoarthritis,” “intervertebral disc disease,” “tendinopathy,” and “low back pain.” Boolean operators (AND/OR) were applied as appropriate. The search covered publications from January 2000 to March 2025, with preference given to recent and methodologically robust studies.

Although the literature search encompassed studies published between January 2000 and March 2025 in order to capture the full evolution of redox biology and ozone-related mechanistic concepts, randomized and controlled clinical trials included in the qualitative synthesis were intentionally restricted to publications from 2020 onward. This temporal restriction was applied to ensure greater methodological homogeneity, consistency in diagnostic criteria, ozone generation technologies, dosing protocols, and outcome assessment tools. Earlier randomized controlled trials frequently employed heterogeneous protocols and non-standardized outcome measures that limited direct comparability with contemporary trials. Therefore, controlled and randomized trials published between 2020 and 2025 were prioritized for the clinical evidence synthesis, whereas older studies were considered primarily for background and mechanistic contextualization. This restriction could have introduced a chronological selection bias: several earlier RCTs with neutral or negative findings were excluded from the clinical synthesis, and their omission may have resulted in a more favorable evidence profile than would be obtained from a fully inclusive analysis. This limitation will be discussed in Section 7.

Eligible articles included basic science investigations, preclinical models, randomized and non-randomized controlled trials, observational studies, and relevant systematic reviews. Case reports, conference abstracts without full text, and studies lacking adequate methodological detail were excluded. Only articles published in English were considered.

**Figure 1:**
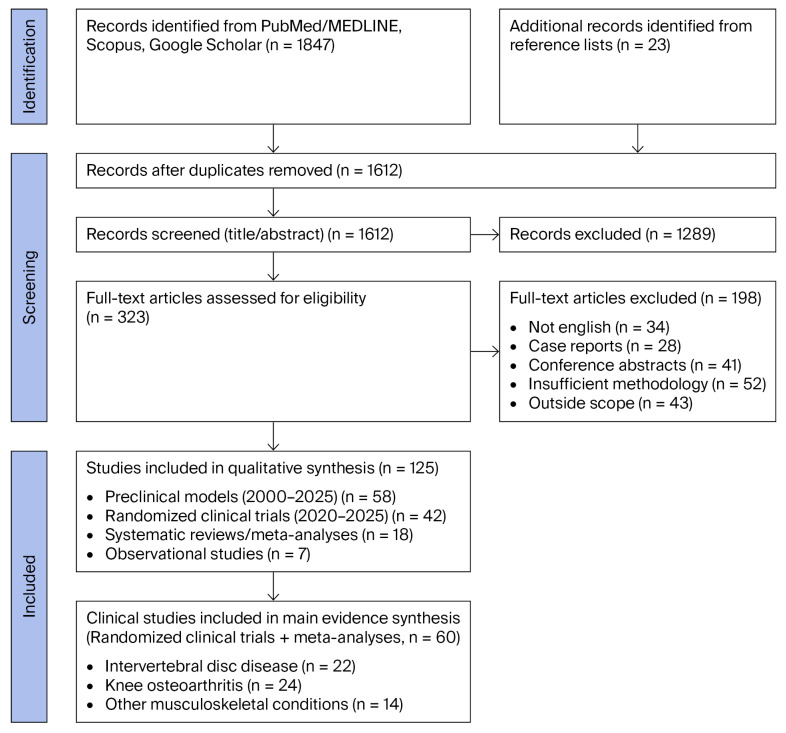
PRISMA flow diagram. Note: PRISMA-style flow diagram illustrating the literature search and study selection strategy employed in this narrative clinical–mechanistic review. Randomized controlled trials were restricted to 2020–2025 to ensure methodological homogeneity; earlier randomized controlled trials (n = 38) were excluded from clinical synthesis but retained for mechanistic contextualization. This diagram documents the search strategy and does not imply a formal systematic review protocol.

Study selection was performed through title and abstract screening, followed by full-text assessment for relevance to the objectives of the review. Data extraction focused on the study design, patient population, musculoskeletal condition, ozone administration route and dosage, comparator interventions, and reported clinical or biological outcomes. Where available, quantitative effect sizes (mean differences, standardized mean differences, relative risks) and 95% confidence intervals were extracted; where original studies did not report these metrics, the absence is noted explicitly in the tables. Owing to heterogeneity across studies, no formal meta-analysis was performed. Evidence was synthesized qualitatively, with an emphasis on biological plausibility, consistency of findings, and methodological limitations, and is presented thematically by mechanistic and clinical domains [9,10]. The PRISMA-style flow diagram shown in Figure 1 documents the search and selection process.

## 3. Redox Biology of Musculoskeletal Degeneration

Degenerative musculoskeletal disorders are increasingly understood as conditions characterized by dysregulated cellular homeostasis rather than purely mechanical tissue failure. Experimental evidence has shown that altered inflammatory and redox signaling is linked to the dysregulation of mechanobiological responses in musculoskeletal tissues. In fact, cells residing in cartilage, intervertebral disc, and tendon structures require finely tuned redox-sensitive pathways to translate mechanical loading into anabolic or reparative signals. Chronic oxidative imbalance disrupts these pathways, leading to impaired mechanotransduction and reduced capacity to adapt to physiological stress, thereby accelerating degenerative changes even in the absence of overt injury [11].

Systemic factors further contribute to redox dysregulation in degenerative musculoskeletal diseases. Metabolic disorders, vascular insufficiency, and age-related decline in antioxidant capacity may amplify local oxidative stress, creating a permissive environment for tissue degeneration. This systemic–local interaction helps explain the multifocal and progressive nature of musculoskeletal degeneration and supports the concept of redox imbalance as a shared pathogenic mechanism across different anatomical sites rather than a tissue-specific phenomenon [12].

According to recent evidence in redox biology, ROS are continuously generated as by-products of aerobic metabolism in human cells, but their concentration is tightly regulated by enzymatic and non-enzymatic antioxidant systems; this controlled redox environment allows ROS to function as signaling molecules involved in cell survival, energy metabolism, and tissue adaptation [13]. In degenerative musculoskeletal diseases, however, sustained inflammatory stimuli, mechanical overload, and age-related mitochondrial dysfunction disrupt this balance, leading to excessive or poorly regulated oxidant production.

Consequently, in cartilage and intervertebral disc tissues, chronic redox imbalance promotes the degradation of extracellular matrix components, suppression of anabolic pathways, and activation of catabolic enzymes such as matrix metalloproteinases. Thus, it has been reported that in the pathophysiological process of musculoskeletal degeneration, oxidative stress contributes to cellular senescence, apoptosis, and the reduced proliferative capacity of chondrocytes and disc cells, ultimately impairing tissue repair and regeneration. Similar mechanisms have been described in tendinopathies, where oxidative stress interferes with collagen production and tenocyte function [14,15].

Importantly, disease progression appears to reflect not only cumulative oxidative damage, but also the failure of adaptive redox-regulatory mechanisms. Studies have reported that the impaired activation of redox-sensitive transcription factors has been associated with reduced cellular resilience and heightened vulnerability to chronic stressors in musculoskeletal tissues, and an increasing amount of evidence supports the pivotal role of nuclear releasing factor 2 (Nrf 2) in coordinating antioxidant defenses, mitochondrial biogenesis, and cytoprotective responses [16,17].

These observations have important therapeutic implications. Interventions aimed exclusively at scavenging free radicals or suppressing inflammation may not adequately restore physiological redox signaling and, in some contexts, may blunt endogenous adaptive responses. Consequently, there is increasing interest in conservative strategies able to modulate redox homeostasis by promoting the controlled activation of cytoprotective pathways, thereby enhancing tissue resilience and functional recovery rather than simply inhibiting oxidative or inflammatory processes. The next paragraphs will elucidate the mechanism of action and the clinical application of ozone in the treatment of musculoskeletal diseases.

## 4. Biochemical Mechanisms of Action of Ozone Therapy

### 4.1. Pharmacological Actions of Medical Ozone

Ozone (O_3_) is a triatomic oxygen molecule usually administered in musculoskeletal diseases as an oxygen–ozone gas mixture. Due to its high reactivity as an oxidant, ozone does not persist as a stable compound in biological systems, and after contact with human fluids, it rapidly reacts with water-soluble antioxidants and polyunsaturated fatty acids, generating a series of secondary oxidative mediators that are responsible for therapeutic effects [18,19,20].

Two principal classes of mediators are generated following tissue’s exposure to ozone: reactive oxygen species (ROS)—primarily hydrogen peroxide—and lipid oxidation products (LOPs), including aldehydes and hydroxyalkenals. Hydrogen peroxide acts as a short-lived intracellular signaling molecule capable of diffusing across cell membranes and modulating redox-sensitive pathways. In contrast, LOPs exhibit longer half-lives and mainly exert systemic effects by interacting with cellular receptors and transcriptional regulators.

At low concentrations, ozone-derived ROS and LOPs are able to stimulate adaptive cytoprotective mechanisms rather than inducing irreversible oxidative injury. These mechanisms of action are largely related to the intracellular activation of redox-sensitive transcription factors, particularly nuclear factor erythroid 2-related factor 2 (Nrf2). In cytoplasma, the oxidative modification of cysteine residues on Kelch-like ECH-associated protein 1 (Keap1) promotes Nrf2 dissociation and nuclear translocation, leading to the transcription of a number of genes involved in antioxidant defense, detoxification processes, and the maintenance of cellular redox balance. Upregulated gene products include enzymes regulating glutathione synthesis and recycling, thioredoxin systems, nicotinamide adenine dinucleotide phosphate (NADPH) regeneration, and heme oxygenase activity. Collectively, these pathways contribute to enhanced cellular resilience, improved mitochondrial function, and the modulation of inflammatory signaling cascades. Importantly, this response to ozone-derived ROS and LOP messengers is transient and dose-dependent, underscoring the importance of calculated ozone concentration and administration protocols [21].

Beyond Nrf2 activation, ozone-derived mediators have also been shown to influence additional redox-sensitive pathways involved in inflammation and cellular metabolism. Experimental data suggest the modulation of nuclear factor kappa B (NF-κB) signaling, with downstream effects on pro-inflammatory cytokine expression and a modulation of immune cell function. In parallel, transient oxidative signaling may influence mitochondrial dynamics, promoting biogenesis and improving oxidative efficiency, thereby reducing baseline reactive oxygen species generation over time [8].

It is important to highlight that the biological response to ozone exposure appears to be context-dependent, influenced by baseline redox status, tissue oxygenation, and inflammatory burden. Consequently, cells with impaired adaptive capacity may exhibit exaggerated responses to oxidative stimuli, whereas tissues with preserved redox flexibility may respond with enhanced cytoprotective activation. This variability emphasizes the importance of individualized treatment protocols and careful patient selection, particularly in degenerative musculoskeletal conditions characterized by heterogeneous disease phenotypes.

Based on these considerations, ozone therapy should therefore not be interpreted as an exogenous antioxidant treatment or a direct anti-inflammatory intervention. Instead, its proposed mechanism relies on the induction of a transient and controlled oxidative challenge able to stimulate endogenous adaptive responses. This distinction is critical for understanding both the potential therapeutic effects and the risks associated with inappropriate dosing or non-standardized application in musculoskeletal disorders.

### 4.2. Hormesis and Therapeutic Redox Modulation

The therapeutic effects attributed to ozone should be interpreted according to the concept of hormesis—a dose–response phenomenon in which low-intensity stressors elicit adaptive and protective cellular responses, but excessive exposure results in tissue injury. In redox biology, hormesis reflects the dual role of reactive oxygen species as both signaling mediators and potential sources of oxidative damage [22].

As previously reported, ozone-derived reactive oxygen species and lipid oxidation products are transient signals able to activate endogenous defense mechanisms within cells. Experimental evidence indicates that such mild oxidative challenges enhance mitochondrial efficiency, upregulate antioxidant enzyme systems, and improve cellular tolerance to subsequent stressors. Conversely, excessive oxidative stimuli may disrupt redox homeostasis and negate potential benefits, underscoring the narrow therapeutic window of ozone-based interventions.

This hormetic model has important clinical implications for degenerative musculoskeletal diseases, where impaired adaptive capacity and defective stress-response signaling appear to contribute more to disease progression than acute oxidative injury. Therapeutic strategies that promote the controlled activation of redox-sensitive pathways may therefore support tissue resilience and functional recovery. Within this context, ozone therapy represents a potential redox-modulating approach whose effects are critically dependent on dose, route of administration, and patient-specific factors [23].

The concept of hormetic redox modulation also provides a theoretical model for understanding the variability in clinical outcomes reported across ozone therapy studies. Differences in ozone concentration, treatment frequency, and route of administration may shift the biological response along the hormetic curve, resulting in either adaptive benefit, neutral, or toxic effects. This means that therapeutic efficacy is not solely dependent on the presence of an oxidative stimulus, but on its precise calibration within a narrow biological window [24].

## 5. Clinical Applications of Ozone Therapy in Intervertebral Disc Pathology

Intervertebral disc degeneration and herniation are characterized by progressive extracellular matrix breakdown, loss of proteoglycans, and inflammatory changes that contribute to the mechanical instability of the column and radicular pain. Chronic oxidative stress plays a contributory role in these processes through the activation of matrix-degrading enzymes, inhibition of anabolic signaling, and induction of apoptosis and senescence in nucleus pulposus and annulus fibrosus cells, ultimately impairing disc homeostasis and repair capacity [25,26].

Within this pathophysiological landscape, ozone therapy has been applied as a minimally invasive conservative intervention, most commonly via intradiscal or paravertebral administration. Clinical evidence derives from randomized controlled trials, prospective cohort studies, and large observational series evaluating ozone injections in patients with contained disc herniation and limited neurological deficits, in particular. Across these studies, intradiscal ozone therapy has been associated with significant reductions in radicular pain and disability scores, with outcomes that are generally comparable to those achieved with corticosteroid-based or other conservative pharmacological interventions in the short to medium term. Comparative studies suggest that while corticosteroids may provide more rapid early symptom relief, ozone therapy may offer a more sustained clinical benefit over follow-up, potentially reflecting differences in underlying mechanisms of action. Proposed effects of ozone include oxidation-induced reduction in proteoglycan content, leading to decreased disc volume and intradiscal pressure, as well as the modulation of periradicular inflammatory responses through redox-sensitive signaling pathways (Table 1a,b) [27,28,29,30,31,32,33,34,35,36].

Paravertebral administration, used either alone or as an adjunct to intradiscal injection, is thought to contribute additional analgesic and anti-edematous effects by influencing local cytokine release, muscle tone, and microcirculation. Hence, clinical protocols integrating ozone with corticosteroids or local analgesic interventions have also been explored in the paravertebral muscular injection of ozone, with the aim of coupling the rapid anti-inflammatory effects of pharmacological agents with the proposed mechanical and biological actions of ozone. Although such approaches may enhance early pain control, the available data do not consistently demonstrate clear superiority over ozone monotherapy at mid-term follow-up (Table 2 and Table S1) [37,38,39,40,41,42,43,44,45].

Overall, intradiscal ozone therapy appears to represent the most evidence-based viable option within the conservative management spectrum for selected patients, although ozone concentration and injection technique need to be standardized.

## 6. Clinical Application of Ozone Therapy in Knee Osteoarthritis

### 6.1. Knee Osteoarthritis

Degenerative knee osteoarthritis is associated with chronic low-grade inflammation, progressive cartilage degradation, subchondral bone remodeling, and synovial dysfunction, processes in which oxidative stress and altered redox signaling play a key role. In this pathophysiologic condition, increased production of reactive oxygen species within the joint microenvironment has been linked to chondrocyte senescence, reduced extracellular matrix synthesis, and the amplification of pro-inflammatory cytokine pathways, thereby accelerating structural and functional deterioration [46,47].

Intra-articular ozone therapy has been investigated as a conservative intervention in knee osteoarthritis, primarily aimed at symptom control rather than structural modification. Randomized clinical studies report significant improvements in pain, joint stiffness, and functional scores following repeated low-dose ozone injections, particularly in patients with mild-to-moderate disease. Proposed mechanisms include the modulation of synovial inflammation, transient activation of endogenous antioxidant protection systems, and improvement of local microcirculation [48,49].

Comparative studies have evaluated ozone therapy against established intra-articular treatments such as corticosteroids, hyaluronic acid, and platelet-rich plasma (PRP). Corticosteroid injections are often associated with rapid pain relief, but their effects tend to diminish over time, whereas ozone therapy appears to produce a more gradual improvement with sustained benefits at short- to mid-term follow-ups. Comparisons with hyaluronic acid generally indicate comparable clinical outcomes in terms of pain reduction and functional improvement, although differences in injection protocols and outcome measures limit direct equivalence. More recent studies comparing ozone with PRP suggest that both interventions can significantly improve symptoms, with PRP potentially offering longer-lasting effects in selected patients, particularly in earlier stages of osteoarthritis. However, ozone therapy has been associated with simpler administration, lower procedural complexity, and reduced cost (Table 3) [50,51,52,53,54,55,56,57,58,59].

On the whole, the available clinical evidence supports ozone therapy as a potential alternative or adjunctive intra-articular option for the symptomatic management of knee osteoarthritis. Nonetheless, heterogeneity in ozone concentrations, treatment schedules, and follow-up duration remains substantial, underscoring the need for standardized head-to-head trials to better define comparative effectiveness, durability of response, and optimal patient selection.

### 6.2. Use of Ozone in Other Degenerative Musculoskeletal Diseases

Beyond intervertebral disc disease and knee osteoarthritis, ozone therapy has been investigated in a range of other degenerative and overuse-related musculoskeletal conditions, including hip osteoarthritis, hand osteoarthritis, and lateral epicondylitis, as well as shoulder tendinopathies and myofascial pain syndromes [60,61,62]. Despite differences in anatomical location and biomechanical loading, these disorders share common pathophysiological features such as localized inflammation, microvascular dysfunction, and increased ROS content within affected tissues.

In hip osteoarthritis, clinical studies evaluating periarticular or intra-articular ozone injections report reductions in pain intensity and improvements in mobility and functional scores, with outcomes comparable to other conservative injectable therapies in short-term follow-up [63]. Hand osteoarthritis has been explored in smaller randomized and observational studies, in which local ozone administration was associated with pain reduction and improvements in hand function and grip strength, particularly in patients with symptomatic interphalangeal joint involvement [64]. In lateral epicondylitis, ozone therapy has been assessed as an alternative to corticosteroid injections, with reported benefits in pain reduction and functional recovery, especially at mid-term follow-up [65].

Additional investigations in shoulder disorders, including rotator cuff tendinopathy and adhesive capsulitis, suggest that ozone injections may reduce pain and improve range of motion, particularly when combined with physiotherapy or exercise-based rehabilitation. Although these findings are encouraging, the overall evidence base for these indications remains limited by small sample sizes, heterogeneity in treatment protocols, and short follow-up durations. Consequently, ozone therapy in these conditions should be regarded as an adjunctive option within conservative management strategies, pending confirmation through larger, condition-specific randomized controlled trials [66].

## 7. Discussion

### 7.1. From Mechanistic Plausibility to Clinical Evidence: Bridging the Gap

The body of evidence reviewed here points to a consistent, if circumscribed, conclusion: in patients with degenerative musculoskeletal disease, ozone therapy administered within validated concentration ranges produces short- to mid-term reductions in pain and disability that are broadly comparable to those achieved with established conservative interventions. This convergence of findings across anatomically and pathophysiologically distinct conditions—lumbar disc herniation, knee osteoarthritis, and several overuse-related disorders—lends biological credibility to the hypothesis that a shared mechanism, i.e., redox modulation, may underlie the observed clinical benefits. Yet the critical distance between mechanistic plausibility and demonstrated efficacy remains substantial, and it is this gap that most limits the current appraisal of ozone therapy.

As a result, the mechanistic narrative, however compelling experimentally, remains unvalidated at the clinical level.

#### A Proposed Translational Protocol: From In Vitro to Clinic

The preclinical support for ozone therapy derives largely from experimental models demonstrating Nrf2 nuclear translocation, the upregulation of glutathione-related enzyme systems, and the modulation of NF-κB-dependent signaling following controlled ozone exposure [20,21]. These findings are internally coherent and consistent with the hormetic mechanism of action. However, the translation of this model into clinical benefit rests on an inference that has never been directly tested in human tissue at clinically administered doses. A staged translational protocol to bridge this mechanistic gap is proposed below.

Stage 1 (In Vitro): Standardized cell-line studies using human chondrocytes, nucleus pulposus cells, and tenocytes exposed to clinically relevant ozone concentrations (10–40 µg/mL) and contact times. Endpoints should include validated Nrf2 translocation assays (immunofluorescence or Western blot), glutathione redox ratio (GSH/GSSG), and downstream antioxidant enzyme expression (HO-1, NQO1, GPx).

Stage 2 (Ex Vivo): Human tissue explant models to validate matrix turnover markers (MMP-3, MMP-13, ADAMTS-4, aggrecan) under controlled oxidative challenge.

Stage 3 (In Vivo): Animal models with standardized protocols, longitudinal biomarker assessment, and structural imaging.

Stage 4 (Clinical): Dose escalation and efficacy trials, but only after mechanistic validation at Stages 1–3.

Previous literature has largely bypassed Stage 1 standardization, leaping from theoretical redox biology directly to clinical application. This creates a significant mechanistic gap that the field must address before ozone therapy can be positioned as an evidence-based intervention.

### 7.2. Protocol Heterogeneity as a Structural Barrier to Synthesis

Protocol heterogeneity in ozone therapy research represents more than a methodological inconvenience: it is fundamentally incompatible with the hormetic model on the basis of the clinical efficacy of ozone. The trials reviewed here employed ozone concentrations ranging from 10 to 40 µg/mL, with variable injection volumes, differing anatomical targets, and divergent co-interventions—corticosteroids, hyaluronic acid, and exercise programs—applied inconsistently across study arms. Hormesis predicts that the relationship between oxidative stimulus and biological response is nonlinear and threshold-dependent: concentrations below the adaptive zone produce no effect, those within it activate cytoprotective pathways, and those above it cause tissue injury [21,22]. This prediction implies that concentration and dosing precision are not secondary parameters but primary determinants of outcome, yet no trial has explicitly mapped a dose–response curve or stratified patients by baseline redox status to identify where individual subjects fall on the hormetic curve.

This heterogeneity also precludes meaningful cross-study comparisons and renders meta-analytic pooling statistically questionable regardless of effect size estimates. The systematic review conducted by Steppan et al., which pooled nearly 8000 patients across multiple cohorts, demonstrated aggregate effectiveness for intradiscal ozone but also found that complication rates and outcome definitions varied so substantially across centers that aggregate figures may obscure clinically important subgroup differences [36]. More recent meta-analyses confirm pain reduction at the aggregate level while acknowledging that concentration, route, and co-treatment heterogeneity prevent definitive conclusions about optimal protocols [33,34]. Establishing consensus-derived, condition-specific protocols is therefore not merely an academic exercise but a prerequisite for reliable efficacy assessment.

#### Instrumentation and Physical Variables in Ozone Delivery

Protocol heterogeneity in ozone therapy research is fundamentally incompatible with the precision required by the hormetic model. Beyond biological variables, the physical and instrumental parameters of ozone generation and delivery remain critically underreported; ozone generator technology (corona discharge vs. UV-based systems) influences concentration accuracy and stability; and gas mixture ratios (O_2_/O_3_) and injection speed affect the actual dose delivered to tissue. The dynamics of gas dissolution in biological fluids—governed by Henry’s law and local tissue pressure—determine the effective concentration at the cellular level. Without standardized biomedical equipment, calibrated flow meters, and real-time ozone concentration monitoring, “dose” remains an imprecise variable. Consequently, claims of precise hormetic calibration are speculative in the absence of instrument-level standardization. Future trials must report generator type, gas flow rate, mixture concentration, needle gauge, and injection speed to enable reproducible protocol comparisons.

### 7.3. Blinding Challenges and the Contribution of Nonspecific Effects

Blinding is structurally problematic in ozone injection trials. The gas has a recognizable odor detectable by operators, and the tactile and pressure sensations accompanying the injection of a gaseous medium create sensory cues that saline or sham needling cannot plausibly replicate. These constraints have led most trials to use oxygen as the control condition—a design that introduces a distinct confounder, since hyperoxia is itself biologically active: it modulates vascular tone, reduces baseline hypoxia-inducible signaling, and may influence redox-sensitive pathways independently of ozone-specific effects [8,18]. The observed between-group differences in ozone versus oxygen trials may therefore reflect the marginal benefit of ozone-specific oxidative signaling over hyperoxia, a nonspecific response to gaseous injection per se, differential placebo amplification driven by the novelty and invasiveness of the procedure, or some combination of all three. Procedural placebo effects in chronic musculoskeletal pain trials are well documented, often substantial, and rarely adequately quantified in ozone studies.

A scientifically valid sham control for ozone injection trials would include needle insertion at the same anatomical target without gas delivery (dry needling/placebo needle); subtherapeutic inert gas volume (e.g., 0.5 mL versus therapeutic volume) to replicate pressure sensation without pharmacological effect; and objective blinding verification using validated indices (e.g., Bang’s Blinding Index) assessed independently of the treating physician. Practical challenges—including ozone odor detection and operator unblinding—may be mitigated by separate procedure rooms, nasal filters for operators, or independent assessors. Trials comparing ozone to inert sham with objective blinding verification are needed before the specific contribution of ozone can be disentangled from contextual and expectancy effects. Pre-registration, pre-specified analysis plans, and CONSORT adherence are non-negotiable in this context.

### 7.4. Statistical Significance, Clinical Relevance, and the Absence of Structural Endpoints

Many of the reviewed trials report statistically significant reductions in visual analog scale (VAS) scores or disability indices without explicitly reporting whether these reductions meet or exceed the minimal clinically important difference (MCID) for the relevant instrument. For reference, established MCID thresholds in musculoskeletal pain trials include VAS ≥ 1.5–2.0 points; Oswestry Disability Index (ODI) ≥ 10–12 points (or ≥30% improvement); Western Ontario and McMaster Universities Osteoarthritis Index (WOMAC) ≥ 12 points; and Knee Injury and Osteoarthritis Outcome Score (KOOS) ≥ 8–10 points (subscale-dependent). A VAS reduction of 1.5 points, for example, may reach statistical significance in an adequately powered trial while remaining at or below the threshold that patients perceive as meaningful improvement. The absence of systematic MCID reporting in ozone trials makes it difficult to translate statistically positive results into clinical guidance.

More consequentially, no published trial has incorporated structural endpoints—serial MRI-assessed disc hydration indices, quantitative cartilage volume measurements, or subchondral bone integrity scores—as primary or secondary outcomes. Without structural data, ozone therapy cannot be characterized as disease-modifying regardless of pain outcomes. The available evidence supports its characterization as a symptom-modulating intervention in selected patients with relatively preserved tissue architecture and impaired redox-inflammatory signaling; it does not support claims of structural disease modification. Trials of at least 24 months incorporating structural imaging alongside patient-reported outcomes are required before any revision of this assessment is warranted.

### 7.5. Safety Data: Reassuring in Controlled Settings, Incomplete in Real-World Practice

Within the concentration ranges employed in the clinical trials reviewed—predominantly 10–40 µg/mL for intra-articular and paraspinal applications—adverse events were generally mild, transient, and self-limiting. The most commonly reported reactions include injection-site discomfort, transient post-procedural pain exacerbation, localized hematoma, and occasional vasovagal episodes, all resolving without specific intervention. The meta-analysis carried out by Steppan et al. reported a complication rate well below 1% across approximately 8000 patients, a figure that has been broadly corroborated in subsequent controlled series [36]. A review similarly found that seven independent systematic reviews consistently identified a low prevalence of minor adverse events attributable to ozone therapy for knee osteoarthritis [67].

These reassuring aggregate figures, however, must be contextualized carefully. Trial populations are highly selected, follow-up periods are short, and the operator expertise characteristic of academic interventional centers may not be reproducible in routine clinical settings. Small trial samples are statistically underpowered to detect rare but clinically serious events, meaning that the true incidence of severe complications is almost certainly underestimated in the published literature. Serious adverse events have been documented outside the controlled trial setting. Rouhi et al. reported neurological complications—including thalamic and cerebellar MRI signal abnormalities consistent with vascular or oxidative injury—following ozone injections, noting that improper needle placement and inadvertent intravascular injection represent the primary pathophysiological mechanisms [68]. Chirumbolo et al. specifically attributed documented serious complications to protocol deviations and inadequate operator training, emphasizing that the safety profile of ozone therapy is critically contingent on adherence to validated procedures [69]. Epidural abscess, nerve injury, cauda equina syndrome, and pneumoperitoneum following intradiscal or paravertebral injection have been described in case reports and small series, underscoring the fact that serious harm, while uncommon under supervised conditions, is not negligible [70].

A separate, and arguably more pressing, safety concern pertains to the long-term biological effects of repeated oxidative challenges in tissues with limited regenerative capacity [71,72,73]. Cartilage, intervertebral disc, and tendon exhibit minimal intrinsic regenerative potential. Future longitudinal studies must incorporate specific senescence and matrix degradation biomarkers to ensure that serial ozone injections do not accelerate the very tissue deterioration they are intended to prevent. Recommended biomarker panels include the following:

- Senescence markers: p16INK4a, SA-β-galactosidase, telomere length.
- Matrix degradation markers: MMP-3, MMP-13, ADAMTS-4, aggrecan fragments (ARGS).
- Collagen turnover markers: PINP (procollagen type I N-terminal propeptide), CTX-I (C-terminal telopeptide of type I collagen).

The theoretical risk that serial oxidative challenges might accelerate matrix degradation and cellular senescence has never been evaluated in a longitudinal study extending beyond 12–24 months. Acceptable short-term safety in controlled settings must not be conflated with a clean long-term safety record.

Another safety concern pertains to the absence of regulatory standardization. Ozone therapy remains unapproved by the U.S. Food and Drug Administration for musculoskeletal indications, and its regulatory status varies substantially across European, Asian, and Latin American jurisdictions. Major clinical practice guidelines, including those from the American College of Rheumatology (ACR), the Osteoarthritis Research Society International (OARSI), and the American Academy of Orthopaedic Surgeons (AAOS), do not currently recommend ozone therapy for osteoarthritis or other degenerative musculoskeletal conditions. The ACR/Arthritis Foundation 2020 guideline for knee, hip, and hand osteoarthritis does not include ozone therapy among recommended non-surgical interventions. Similarly, the AAOS Clinical Practice Guideline for the management of knee osteoarthritis (third edition, 2022) does not evaluate or endorse ozone therapy. The OARSI 2019 guidelines for the non-surgical management of knee, hip, and polyarticular osteoarthritis similarly do not consider ozone therapy among evidence-based treatment options. This absence of endorsement reflects the current lack of high-quality, long-term RCTs with validated mechanistic endpoints and standardized protocols, rather than evidence of ineffectiveness per se [74,75,76].

### 7.6. Clinical Positioning and Evidence Hierarchy

There is no doubt that ozone therapy occupies a defensible but carefully circumscribed position in musculoskeletal medicine: it is a mechanistically credible, symptomatically useful adjunctive option for patients with mild-to-moderate degenerative disease who have not responded adequately to conventional conservative measures and who wish to defer surgical intervention. This positioning applies most clearly to patients with symptomatic lumbar disc herniation and mild-to-moderate knee osteoarthritis, conditions for which the controlled trial evidence is most extensive. For other anatomical sites—hip, hand, shoulder, and lateral epicondyle—the evidence base is smaller and more heterogeneous, justifying a more cautious interpretive stance.

However, ozone therapy is not, based on current evidence, a disease-modifying treatment or a standalone standard of care for any degenerative musculoskeletal condition. The clinical support for this therapy remains limited by methodological shortcomings across the published trial literature. Publication bias must also be acknowledged: a disproportionate number of clinical trials originate from investigators with established ozone programs or from industry-supported device studies. Independent, publicly funded, multicenter trials conducted under pre-registered protocols represent the only path to a scientifically correct assessment of efficacy.

## 8. Conclusions

Ozone therapy for degenerative musculoskeletal disease rests on a coherent and experimentally grounded biological rationale: controlled, dose-dependent oxidative signaling activates Nrf2-mediated cytoprotective pathways and modulates redox-sensitive inflammatory cascades through a mechanism consistent with the hormetic model. Based on a series of experimental studies, ozone does not function as an exogenous antioxidant or a direct anti-inflammatory agent; rather, it acts as a calibrated redox stimulus capable of restoring adaptive cellular resilience in tissues where endogenous redox-regulatory mechanisms are chronically impaired.

Clinical results, particularly from randomized controlled trials published between 2020 and 2025, indicate that ozone therapy produces short- to mid-term improvements in pain and functional outcomes that are comparable to corticosteroid injections, non-inferior to microdiscectomy in selected patients with lumbar disc herniation, and broadly equivalent to hyaluronic acid for symptomatic knee osteoarthritis over similar follow-up periods. These findings are consistent across multiple anatomical sites and study designs, lending them greater credibility than any single trial could provide in isolation.

Nevertheless, current evidence shows substantial methodological limitations that prevent definitive conclusions. The specific contribution of ozone—as distinct from the nonspecific effects of gaseous injection, procedural expectancy, and active oxygen comparators—has not been adequately isolated in any published trial. No study has validated the proposed mechanism of Nrf2-mediated redox modulation in human tissue at clinically administered doses. Protocol heterogeneity across trials is pervasive and incompatible with the precision required by the hormetic model. Statistically significant outcomes do not consistently exceed MCID thresholds, and structural endpoints documenting disease modification are entirely absent. Short-term safety in controlled, academically supervised settings is acceptable, but the true incidence of serious complications in private clinical practice is unknown, and long-term safety data beyond 24 months are lacking.

Consequently, advances in the field of ozone therapy for musculoskeletal diseases require a systematic and methodologically disciplined research agenda, taking into account the aforementioned constraints.

Until this evidence exists, the clinical adoption of ozone therapy should remain cautious, protocol-driven, and accompanied by transparent informed consent that accurately represents the investigational nature of the intervention. The biological rationale for ozone therapy is sufficiently robust to justify continued rigorous investigation, but it does not support uncritical clinical expansion. Realizing the therapeutic potential of this approach will require the same methodological discipline demanded of any emerging intervention—mechanistic rigor, protocol standardization, and extended follow-up that can clarify lasting benefit and delayed harm.

## Figures and Tables

**Table 1:** (**a**) Randomized clinical trials on intradiscal or paravertebral ozone injection/disc herniation (2020–2025). Individual trials only; (**b**) systematic reviews and meta-analyses of intradiscal ozone therapy (2020–2025).

(**a**)							
**Study**	**Year**	**Design**	**Condition**	**Sample**	**Clinical Outcome**	**Effect Size (95% CI)**	**Follow-Up**
Ercalik T et al. [29]	2020	DB-RCT	Lumbar disc herniation	60	Ozone alone effective; adding steroids conferred no additional benefit	VAS MD −2.1 (95% CI NR)	1 month
Clavo B et al. [31]	2021	DB-RCT	Chronic discogenic LBP	60 vs. 60 vs. 60	Ozone: 20% required surgery vs. 60% in oxygen group	RR 0.33 (95% CI 0.14–0.78)	Median 78 mo
Kelekis A et al. [28]	2022	Non-inferiority RCT	Lumbar disc herniation with radiculopathy	140 vs. 140	Ozone non-inferior to microdiscectomy for pain and disability; lower complication rate	ΔVAS −0.4 (95% CI −1.2 to 0.4)	1 year
Sucuoğlu H et al. [37]	2021	DB-RCT	Acute lumbar disc herniation	38	Paravertebral ozone effective as adjunctive treatment for acute LDH	VAS MD −2.4 (95% CI NR)	1 month
Rayegani SM et al. [30]	2023	RCT	Lumbosacral canal stenosis	50 (25 vs. 25)	Addition of ozone improved outcomes in spinal stenosis	VAS MD −1.8; ODI MD −8.5 (95% CI NR)	1 and 6 mo
Yang Y et al. [32]	2023	DB-RCT	Chronic discogenic LBP	120	Ozone + steroid significantly superior at 3 and 6 months	VAS MD −2.29 at 3 mo; −2.25 at 6 mo (*p* < 0.001)	6 months
Parvin R et al. [38]	2024	DB-RCT	Lumbar spinal stenosis	30	Ozone superior to steroids at 8 weeks (53.8% pain reduction)	VAS MD −3.2 (95% CI NR)	8 weeks
Forogh B et al. [39]	2025	DB-RCT	Chronic discogenic LBP	30 (15 vs. 15)	Ozone–oxygen injection effective vs. pure oxygen control	VAS MD −2.6; ODI MD −6.8 (95% CI NR)	1 and 3 mo
(**b**)							
**Study**	**Year**	**Design**	**Condition**	**Sample**	**Clinical Outcome**	**Pooled Effect Size (95% CI)**	**Follow-Up**
Chang MC et al. [34]	2024	Systematic review and meta-analysis	Herniated lumbar disc	6 RCTs	Intradiscal ozone effective for pain relief; comparable to other interventions	SMD −0.82 (95% CI −1.31 to −0.33)	Various
Cao D et al. [33]	2025	Meta-analysis (8 RCTs)	Lumbosacral pain/disc herniation	1744 total	Ozone significantly reduced pain and disability; superior short-term efficacy	VAS MD −2.13 (95% CI −2.89 to −1.37); ODI MD −0.79 (95% CI −1.24 to −0.34)	Short-term

DB-RCT = double-blind randomized controlled trial; RCT = randomized controlled trial; NR = not reported; VAS = visual analog scale; ODI = Oswestry Disability Index; LBP = lower back pain; RR = relative risk; SMD = standardized mean difference; MD = mean difference; mo = months. Effect sizes extracted or estimated from published reports where available; 95% CIs reported when provided in original studies. MCID thresholds: VAS ≥ 1.5–2.0 points; ODI ≥ 10–12 points (or ≥30% improvement).

**Table 2:** Randomized controlled trials on paravertebral ozone injection (2020–2025). Note: Observational and retrospective studies (e.g., Latini et al., 2024 [43]) are reported separately in Supplementary Table S1.

Study	Year	Design	Condition	Sample	Clinical Outcome	Effect Size (95% CI)	Follow-Up
Sucuoğlu H et al. [37]	2021	DB-RCT	Acute lumbar disc herniation	38	Paravertebral ozone effective as adjunct to conservative management	VAS MD −2.4 (95% CI NR)	1 month
Yang Y et al. [32]	2023	DB-RCT	Chronic discogenic LBP	120	Ozone + steroid superior at 3–6 months vs. steroid alone	VAS MD −2.29 at 3 mo; −2.25 at 6 mo (*p* < 0.001)	6 months
Parvin R et al. [38]	2024	DB-RCT	Lumbar spinal stenosis	30	Ozone superior to steroids at 8 weeks; steroids faster initially	VAS MD −3.2 (95% CI NR)	8 weeks
Forogh B et al. [39]	2025	DB-RCT	Chronic discogenic LBP	30 (15 vs. 15)	Ozone–oxygen injection effective vs. pure oxygen control	VAS MD −2.6; ODI MD −6.8 (95% CI NR)	1 and 3 mo

DB-RCT = double-blind randomized controlled trial; LBP = lower back pain; VAS = visual analog scale; ODI = Oswestry Disability Index; MD = mean difference; mo = months; NR = not reported in original publication. MCID thresholds: VAS ≥ 1.5–2.0 points; ODI ≥ 10–12 points (or ≥30% improvement).

**Table 3:** Randomized controlled trials on intra-articular ozone injection in knee osteoarthritis (2020–2025).

Study	Year	Design	Intervention	Sample	Clinical Outcome	Effect Size (95% CI)	Follow-Up
Farpour HR et al. [50]	2021	DB-RCT	Ozone prolotherapy vs. saline	70	Ozone superior to saline for pain reduction and functional improvement	VAS MD −2.8 (95% CI NR); WOMAC MD −12.4 (95% CI NR)	6 months
Sconza C et al. [52]	2023	DB-RCT	Intra-articular ozone vs. HA (3 injections each)	60	Both improved; ozone showed better cytokine reduction; comparable clinical efficacy	VAS MD −1.9 vs. −1.7 (95% CI NR); between-group *p* = 0.42	6 months
Aslan SG et al. [53]	2024	Multicenter DB-RCT	Ultrasound-guided ozone vs. corticosteroid	82	No significant difference between groups for pain, disability, or function	VAS MD −0.3 (95% CI −1.4 to 0.8); *p* = 0.61	6 months
Nazarieh M et al. [54]	2024	DB-RCT	Intra-articular ozone + exercise vs. placebo + exercise	33 (42 knees)	Ozone significantly superior at 6 weeks, 1 month, and 6 months (pain and KOOS)	VAS MD −2.1 (95% CI NR); KOOS MD +8.3 (95% CI NR)	6 months
Arjmanddoust Z et al. [55]	2025	DB-RCT	Ozone 20 µg/mL vs. 40 µg/mL vs. oxygen control (4 weekly injections)	59	Both ozone doses effective vs. control; no significant difference between concentrations	VAS MD −2.4 (20 µg/mL) and −2.6 (40 µg/mL) vs. −0.8 (control); 95% CI NR	2 months
Raeissadat SA et al. [57]	2021	RCT (4-arm)	Intra-articular ozone vs. HA vs. PRP vs. PRGF	200	Ozone superior short-term (2 months); HA and PRP superior at 6–12 months	VAS MD −3.1 at 2 mo (95% CI NR); HA/PRP MD −4.2 at 12 mo (95% CI NR)	12 months

DB-RCT = double-blind randomized controlled trial; RCT = randomized controlled trial; HA = hyaluronic acid; PRP = platelet-rich plasma; PRGF = platelet-rich growth factor; VAS = visual analog scale; WOMAC = Western Ontario and McMaster Universities Osteoarthritis Index; KOOS = Knee Injury and Osteoarthritis Outcome Score; MD = mean difference; NR = not reported in original publication. MCID thresholds: VAS ≥ 1.5–2.0 points; WOMAC ≥ 12 points; KOOS ≥ 8–10 points (subscale-dependent).

## Data Availability

No new data were created or analyzed in this study. Data sharing is not applicable to this article.

## Supplementary Materials

The following supporting information can be downloaded at: https://www.mdpi.com/article/10.3390/jcm15156065/s1. Table S1: Observational and retrospective studies on paravertebral ozone therapy (2020–2025). Note: These studies were excluded from the main RCT synthesis due to non-randomized design.
