# Supplementary material for: Ozone Therapy as a Redox-Modulating Strategy in Degenerative Musculoskeletal Diseases: A Clinical–Mechanistic Review
Source: J Clin Med. 2026 Aug 4;15(15):6065. doi: 10.3390/jcm15156065 (PMC13467045; doi:10.3390/jcm15156065)
Supplement: Supplementary file 1 [file jcm-15-06065-s001.zip › jcm-4404308-supplementary.pdf]

Table S1. Observational and retrospective studies on paravertebral ozone therapy (2020–2025). Note: These studies were excluded from the main RCT synthesis due to non-randomized design.

Study	Year	Design	Condition	Sample	Clinical Outcome	Effect Size (95% CI)	Follow-Up
Latini E, et al.	2024	Retrospective clinical evaluation	Chronic neck/LBP	76	Safe; sustained benefit over 6-month follow-up	VAS MD −2.2 (95% CI NR); NRS MD −1.8 (95% CI NR)	6 months

LBP = Low Back Pain; VAS = Visual Analog Scale; NRS = Numeric Rating Scale; MD = Mean Difference; NR = Not Reported.
